# Bioglass-Incorporated Methacrylated Gelatin Cryogel for Regeneration of Bone Defects

**DOI:** 10.3390/polym10080914

**Published:** 2018-08-14

**Authors:** Song Kwon, Seunghun S. Lee, A. Sivashanmugam, Janet Kwon, Seung Hyun L. Kim, Mi Yeon Noh, Seong Keun Kwon, R. Jayakumar, Nathaniel S. Hwang

**Affiliations:** 1 School of Chemical and Biological Engineering, the Institute of Chemical Processes, Seoul National University, Seoul 08826, Korea; skwon2675@gmail.com; 2 Interdisciplinary Program in Bioengineering, Seoul National University, Seoul 08826, Korea; lsean@snu.ac.kr (S.S.L.); jhkwon929@gmail.com (J.K.); lucykim619@gmail.com (S.H.L.K.); mno9211@snu.ac.kr (M.Y.N.); otolarynx@snuh.org (S.K.K.); 3 Center for Nanosciences and Molecular Medicine, Amrita Institute of Medical Sciences and Research Center, Amrita Vishwa Vidyapeetham, Kochi 682041, India; sivasa19619@aims.amrita.edu (A.S.); rjayakumar@aims.amrita.edu (R.J.); 4 Department of Otorhinolaryngology—Head and Neck Surgery, Seoul National University Hospital, Seoul 03080, Korea; 5 BioMAX/N-Bio Institute, Seoul National University, Seoul 08826, Korea

**Keywords:** methacrylated gelatin, bioglass, hydrogel, cryogel, bone tissue engineering

## Abstract

Cryogels have recently gained interest in the field of tissue engineering as they inherently possess an interconnected macroporous structure. Considered to be suitable for scaffold cryogel fabrication, methacrylated gelatin (GelMA) is a modified form of gelatin valued for its ability to retain cell adhesion site. Bioglass nanoparticles have also attracted attention in the field due to their osteoinductive and osteoconductive behavior. Here, we prepare methacrylated gelatin cryogel with varying concentration of bioglass nanoparticles to study its potential for bone regeneration. We demonstrate that an increase in bioglass concentration in cryogel leads to improved mechanical property and augmented osteogenic differentiation of mesenchymal cells during in vitro testing. Furthermore, in vivo testing in mice cranial defect model shows that highest concentration of bioglass nanoparticles (2.5 *w*/*w* %) incorporated in GelMA cryogel induces the most bone formation compared to the other tested groups, as studied by micro-CT and histology. The in vitro and in vivo results highlight the potential of bioglass nanoparticles incorporated in GelMA cryogel for bone regeneration.

## 1. Introduction

Bone regeneration following surgical removal of bone and injuries from sports, trauma, and disease is a challenging obstacle in clinical treatment because it can easily change course and impair the patient’s quality of life. To augment bone regeneration, autografts and allografts are widely used to treat bone defects [1,2], and they are often applied clinically via the Masquelet technique [3] and the Reamer-Irrigator-Aspirator system [4], either independently or in combination. Although using bone grafts during these procedures possess desirable attributes, such as molecular cues, osteogenic cells, and niche for bone regrowth, retrieving these bone grafts still requires invasive and possibly fatal surgeries [5]. Using allografts could compensate for morbidity, but there is the possibility of transferring diseases from the donors [1,6]. In this aspect, application of synthetic biomaterial-based scaffold designed to emulate native bone structure is one of the most viable options for clinicians [7,8,9,10,11,12,13,14]. An effective biomaterial-based scaffold must be biocompatible and biodegradable while possessing adequate mechanical strength, highly interconnected pores, and optimal pore size [10,15]. As a type of scaffold that fulfills these criteria, cryogel-based scaffolds are being studied. Due to its highly interconnected and macroporous structure, cryogels have been investigated as a proper candidate for transportation of cells [16,17].

Materials for cryogel scaffolds vary greatly depending on their functions and characteristics. Synthetic polymers are a type of cryogel scaffold material that is often preferred over others. This includes polyacrylamide [18] and poly(vinyl alcohol) (PVA) [19] because their degradation rates can be manipulated. However, they are mostly biologically inert and lack cell binding moieties. For this reason, studies that develop new scaffolds focus on biological moieties, such as naturally derived polymers [16,20,21,22,23]. Among the various natural polymers, a collagen derivative called gelatin has favorable biological characteristics, such as the Arg-Gly-Asp (RGD) sequence. Having the RGD sequence present in scaffolds is valuable as it promotes cell adhesion, cell migration, and differentiation and helps to formulate extracellular matrix (ECM) similar to that of the bone [24,25]. Unfortunately, unmodified gelatin hydrogel lacks mechanical integrity, so many strategies are employed to prepare a mechanically stable hydrogel. One strategy is to add chemical moieties, such as furan [26,27], methacrylate [28], and others, to not only increase mechanical stiffness but also prepare photocrosslinked gelatin hydrogels [29,30]. There are a plethora of chemical moieties available today. However, methacrylation of gelatin (GelMA) has been widely studied as a photopolymerizable hydrogel because the mechanical property and porosity of GelMA-based scaffold can be controlled by varying the degree of methacrylic group substitution during the GelMA synthesis [24]. Nonetheless, using solely methacrylated gelatin in scaffolds is insufficient to induce osteogenic differentiation in stem cells.

Bioglass (SiO_2_-CaO-P_2_O_5_), like other calcium phosphates, has mustered attention in bone tissue engineering due to its osteoinductivity, biocompatibility, and ability to form strong bonds with bone and soft tissues [31,32]. The Si^4+^ and Ca^2+^ ions in bioglass have notable influence over cell differentiation and regulation of the cell cycle, making them vital, desirable ions for scaffold fabrication [33,34,35]. Moreover, bioglass can strongly bond with host tissue via formation of hydroxyapatite on its surface through surface chemistry under physiochemical conditions [10,36]. However, bioglass cannot be used alone as implants because it is too fragile; instead, it can be amalgamated with biopolymers to create composite scaffolds that possess bioactivity and plasticity [37]. Qu et al. suggested one solution for bioglass, which involved combining gelatin and bioglass hybrid scaffolds for the odontogenic differentiation of human dental pulp stem cells [38].

**Figure 1:**
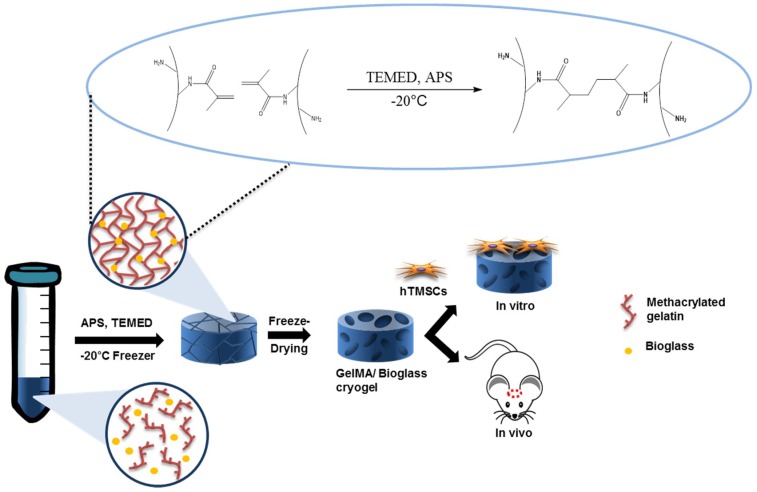
Overall scheme of the nanobioglass-embedded methacrylated gelatin (GelMA) cryogel experiment.

In this study, we investigated the bioactivity of nanobioglass on bone regeneration by fabricating bioglass-embedded methacrylated gelatin-based cryogel. To the best of our knowledge, there has been no prior report on methacrylated gelatin-based cryogel with bioglass—which would bring together the synergistic effect of cryogel, ECM for cell proliferation and host cell infiltration [16,39,40], and bioglass as a bioceramic—for enhancement of mineralization and osteogenic differentiation. We evaluated the mechanical strength, internal structure, cytotoxicity, and osteogenic differentiation ability and further carried out bone regeneration study in mouse cranial defect model. Herein, we demonstrate that nanobioglass-embedded GelMA cryogel induces osteogenic differentiation of human tonsil-derived stem cells (hTMSCs) by observing upregulation of osteogenic genes, calcium deposition, and enhanced bone regeneration in vivo study (Figure 1).

## 2. Materials and Methods

### 2.1. Synthesis of Methacrylated Gelatin

GelMA was synthesized by first dissolving type A porcine skin gelatin (Sigma-Aldrich, St. Louis, MO, USA) into Dulbecco’s phosphate buffer saline (DPBS; Sigma-Aldrich, St. Louis, MO, USA) at 60 °C for 1 h to reach 10% (*w*/*v*) uniform solution [24,41]. An 8% (*v*/*v*) of methacrylic anhydride (Sigma-Aldrich, St. Louis, MO, USA) was then added to 10% (*w*/*v*) gelatin solution at a rate of 0.5 mL/min under stirred condition and reacted for 3 h at 50 °C. The resulting solution was 5×-diluted with warm Dulbecco’s phosphate buffer saline (Gibco, Waltham, MA, USA) and dialyzed using 12–14 kDa cutoff dialysis tube (Sigma-Aldrich, St. Louis, MO, USA) in water for 7 days at 40 °C. The dialyzed solution was placed at −80 °C for a day and lyophilized for another 7 days. After lyophilization, white, foam-type sponge was collected and stored at −20 °C for further use.

### 2.2. Preparation of Nanobioglass

Nanobioglass (SiO_2_:CaO:P_2_O_5_ (mol) = 55:40:5) was prepared by the following protocol [42], with modifications. Briefly, 9.826 mL of tetraethyl orthosilicate (TEOS) diluted in 60 mL of EtOH was added to 120 mL of Ca(NO_3_)_2_·4H_2_O solution (270 mM). The pH of the solution was adjusted to 1–2 with citric acid, and the reaction mixture was stirred vigorously for 12 h. Homogeneous solution was added dropwise to 1500 mL of ammonium dibasic phosphate solution (5.44 mM). During this step, the pH of the solution was maintained at 11 using ammonium hydroxide. The mixture was stirred for 48 h and aged for 24 h, following which the precipitate was separated by centrifugation. The precipitate was washed in EtOH and three times in water. The precipitate was suspended in 2% PEG-water solution and stirred for 30 min. It was freeze-dried for 48 h and then sintered at 700 °C for 3 h. The annealed bioglass was stored at room temperature until further use.

### 2.3. Fabrication of GelMA-Bioglass Cryogel

10 % (*w*/*v*) lyophilized GelMA was fully dissolved in DPBS (Sigma-Aldrich, St. Louis, MO, USA) at 60 °C, and 0.5, 1.5, 2.5 wt % of bioglass were added to GelMA dissolved solution. For each solution, 0.5% (*w*/*v*) of ammonium persulfate (Sigma-Aldrich, St. Louis, MO, USA) and 0.25% (*v*/*v*) of *N*,*N*,*N*′,*N*′-tetramethylethylenediamine (Sigma-Aldrich, St. Louis, MO, USA) were added to initiate polymerization. Then, the solutions were pipetted to 200 µL mold (cylindrical shape—diameter: 8 mm and height: 3 mm) and placed at −20 °C for 24 h to slow down polymerization reaction while maximizing ice crystal fragments for larger pores. After gelation, ice fragments were removed via lyophilization, and cryogels were obtained. Cryogels were stored at −80 °C for further use.

### 2.4. Swelling Ratio and Mechanical Property of Scaffold

Swelling ratio test was performed to investigate water retention of cryogels according to bioglass concentration. For swelling tests, dry weights of each cryogel were measured and transferred to DPBS (Sigma-Aldrich, St. Louis, MO, USA) for a day to swell. After 24 h, cryogels in DPBS were collected, and swelling weights of each cryogels were measured. The average swelling ratios of cryogels were calculated based on the following equation:

Swelling ratio = $\frac{W_{s}}{W_{d}}$, where *W_s_* is the swell weight of cryogels and *W_d_* is dried weight of cryogels.

Cryogels were swollen in DPBS for 24 h and tested for Young’s modulus using the universal testing machine (Universal testing machine, EZ-SX, Shimadzu, Kyoto, Japan). Gels were compressed with the loading rate of 1 mm/min. The result was obtained by the stress–strain curve, and Young’s modulus was calculated by the measurement of the slope linearly increased in the region of the stress–strain curve. The dataset was then analyzed using the equation:

Young’s modulus = $\frac{\sigma }{\varepsilon }$, where σ represents stress and ε represents strains.

### 2.5. Degradation by Collagenase

Degradation rates of 10% (*w*/*v*) GelMA cryogels, GelMA-0.5% (*w*/*v*) bioglass cryogels, GelMA-1.5% (*w*/*v*) bioglass cryogels, and GelMA-2.5% (*w*/*v*) bioglass cryogels were measured by placing them in 1 unit/mL of collagenase II solution (Worthington Biochemical). Samples were incubated in 37 °C, and old collagenase solutions were replaced with new collagenase solutions every day. Cryogels were removed from the collagenase solutions, and water on the surface of cryogels was removed before swollen weights of cryogel were measured at a time point.

### 2.6. Ion Release Analysis

Bioglass powder was autoclaved before measuring ion release level to remove any contaminants. Then, GelMA cryogels, GelMA-0.5% bioglass cryogels, GelMA-1.5% bioglass cryogels, and GelMA-2.5% bioglass cryogels were fabricated (n = 3) and placed at 24 well plates and filled with 1 mL of simulated body fluid solution containing 58.43 g of NaCl, 2.77 g of CaCl_2_, and 1.39 g of NaH_2_PO_4_·H_2_O (all chemicals from Sigma-Aldrich, St. Louis, MO, USA) in 1 L of deionized water (Sigma-Aldrich, St. Louis, MO, USA) as prepared. The solution was collected at day 1, 3, 5, and 7 and centrifuged at 4000 rpm for 30 min. Solutions were then filtered using 200 nm pore sizes of syringe membrane (Acrodisc^®^) after samples were collected. Ion release rate of Ca^2+^, Si^4+^, and P^3+^ ions were measured with inductively coupled plasma atomic emission spectrometer (ICP-AES, Optima 8300, PerkinElmer, Waltham, MA, USA). To measure ion release rates of bioglass-embedded cryogels in deionized water, the same protocol was used.

### 2.7. Scanning Electron Microscopy

Internal structures of cryogels with different concentration of bioglass were observed via scanning electron microscopy (FE-SEM, JSM-6701F, JEOL, Tokyo, Japan). Cross sections of cryogels were fixed on mounts and coated with platinum at 20 mA for 100 s. Then, internal structures were analyzed by Field emission electron microscopy (FE-SEM; JSM-6701F, JEOL, Tokyo, Japan).

### 2.8. Cell Cultures

Human tonsil-derived mesenchymal stem cells were isolated from tonsillar tissue, which were provided by the Department of Otorhinolaryngology—Head and Neck Surgery, Seoul National University Hospital (Seoul, Korea) with prior consent. The Institutional Review Board (IRB) of Seoul National University Hospital approved this study. For proliferation, hTMSCs were cultured in general medium—10% fetal bovine serum (Biowest), 1% L-glutamine (200 mM) (Sigma), 1% penicillin/streptomycin (10,000 U/mL) (Gibco), and 1% antibiotic-antimycotic (100X; 15240062) (Gibco) in Dulbecco’s modified Eagle’s medium (Gibco). The culture medium was changed every other day.

### 2.9. Cell Viability

Cell viability was observed via Live/Dead viability kit (InvitrogenTM, Waltham, MA, USA). 5 × 10^5^ cells of hTMSCs were seeded per cryogels and were incubated for 24 h. After 24 h, cryogels were washed with DPBS three times and 1 mL of DPBS, including 2 µL calcein AM and 1 µL ethidium homodimer-1, was added to each cryogel. Cryogels were incubated for 30 min and was measured by confocal microscope (Confocal Laser Scanning Microscope, LSM 720, Carl Zeiss, Oberkochen, Germany)

### 2.10. In Vitro Osteogenic Differentiation

hTMSCs were cultured in a 24-well plate until cells were 100% confluent. Then, cells were treated with osteogenic medium, the solution of 10 mM glycerol-2-phosphate (Sigma), 1% of 2-Phospho-L-ascorbic acid (Sigma), 1% dexamethasone (Sigma), 10% fetal bovine serum (Biowest), and 1% penicillin/streptomycin (10,000 U/mL) (Gibco) in Dulbecco’s modified Eagle’s medium. For bioglass-treated groups, osteogenic medium with 0.5%, 1.5%, and 2.5% bioglass concentration was used. The culture medium was changed every other day.

### 2.11. Real Time-PCR

RNAs were extracted from the cell-laden GelMA and GelMA-bioglass cryogel (n = 3) with Trizol (Life Technology, Waltham, MA, USA). The concentration of RNA that was extracted was measured by NanoDrop spectrometer (ND-2000; NanoDrop Technologies) and reverse-transcribed into cDNA using TOPscriptTMReverse Transcriptase Kit (Enzynomics). Real time-PCR was performed using ABI StepOnePlusTM real-time PCR system (Applied Biosystems). cDNA samples were analyzed for relative gene expression of *GAPDH*, *OCN*, *Collagen I*, and *Runx2* when *GAPDH* was used as a house-keeping gene. Relative gene expressions of interests were calculated using –2^ΔΔ*C*t^ method. Primer sequences that were used in RT-PCR were: *GAPDH* (Forward: 5′-CGC TCT CTG CTC CTC CTG TT-3′, Reverse: 5′-CCA TGG TGT CTG AGC GAT GT-3′), *OCN* (Forward: 5′-GCC TTT GTG TCC AAG C-3′, Reverse: 5′-GGA CCC CAC ATC CAT AG-3′), *Collagen I* (Forward: 5′-GTC ACC CAC CGA CCA AGA AAC C-3′, Reverse: 5′-AAG TCC AGG CTG TCC AGG GAT G-3′), *Runx2* (Forward: 5′-ACT GGG CCC TTT TTC AGA-3′, Reverse: 5′-GCG GAA GCA TTC TGG AA-3′).

### 2.12. Alizarin Red Staining

After 21 days of differentiation, cells were washed with DPBS twice and fixed using 4% paraformaldehyde for 15 min at room temperature. Fixed cells were stained with 2% alizarin red staining solution for 20 min and washed three times with distilled water for 5 min each. 

### 2.13. Calvarial Defect Surgical Procedure

All experiments were carried out in accordance with the Guide for the Care and Use of Laboratory Animals by Seoul National University (Approval No. SNU-141229-3-6). All operations were performed under Zoletil 50 (Virbac, Carros, France) and Rompuninj (Bayer, Leverkusen, Germany) anesthesia to minimize animal suffering. Twelve female balb-C mice (OrientBio Co., Seoul, Korea) were used for calvarial defects. Mice were caged and handled in a sterile room at 22 °C and 50% humidity with 12 h of light and dark cycles. Before calvarial defect surgery, mice were under anesthesia via intraperitoneal injection. Under anesthesia, incision was made on forehead, and 4-mm diameter of calvarial defect was performed using a trephine bur attached to hand drilling machine. Cryogels were transplanted to defected sites, and mice were collected after eight weeks of transplantation.

### 2.14. Microcomputed Tomography Analysis

Defected sites of mice were collected and fixed with 4% paraformaldehyde solution. Images of surgical sites were obtained using Skyscan 1172 at 59 kV of operation source voltage, 167 μA of source current, and 40 ms of an exposure time. The projected images were reconstructed into 3D images for further analysis using ReCon MicroCT from Skyscan.

### 2.15. Histological Analysis

After fixing defected area and surrounding tissue of skulls in 4% paraformaldehyde solution for 24 h, skulls were decalcified using 14% ethylene diaminetetraacetic acid (EDTA) at pH 7.4 for 4 days. Then, skulls were embedded in paraffin solutions and longitudinally sectioned at a thickness of 5 µm. Sectioned samples were deparaffinated using xylene solution and gradually washed with tap water. Samples were stained with H&E staining and Masson’s trichrome (MTC) staining and analyzed using light microscope (Olympus, Tokyo, Japan).

### 2.16. Statistical Analysis

Quantitative data in this paper are presented in mean ± standard deviation. The statistical significance was determined using one-way analysis of variance (ANOVA) with * *p* < 0.05, ** *p* < 0.001, and *** *p* < 0.0001.

## 3. Results

### 3.1. Synthesis of Methacrylated Gelatin and Bioglass

**Figure 2:**
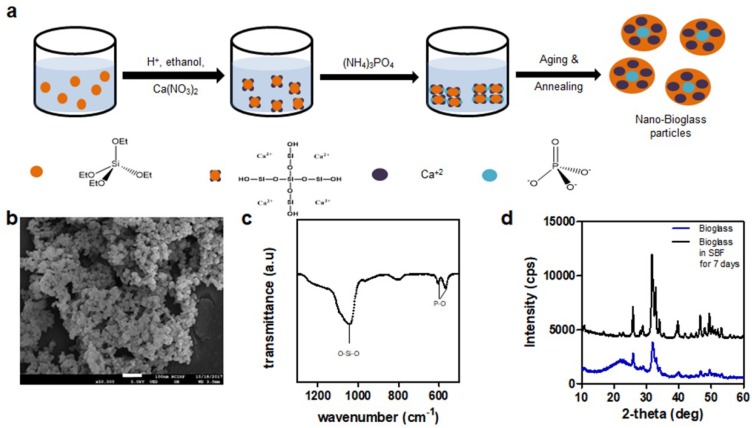
Bioglass characterization (**a**) Schematic representation of nanobioglass synthesis; (**b**) SEM images of bioglass nanoparticle; scale bar = 200 nm; (**c**) FTIR spectrum; (**d**) XRD of bioglass nanoparticle and bioglass nanoparticle immersed in simulated body fluid (SBF) for seven days.

Gelatin methacrylate was synthesized to provide cross-linking sites to conventional gelatin because it required glutaraldehyde, which was reported to be cytotoxic for forming permanent chemical cross-linking. Methacrylation of gelatin was confirmed using ^1^H NMR, as the characteristic peaks of acrylic hydrogen were present at 5.3 and 5.5 ppm (Figure S1). Then, bioglass nanoparticles (BGN) were prepared by sol–gel synthesis route (Figure 2a). The size of bioglass was measured using scanning electron microscopy (SEM). As shown in Figure 2b, the morphology of synthesized bioglass particles was round, amorphous, and around 53.83 ± 13.01 nm in size. The Fourier-transform infrared spectroscopy (FT-IR) was used to further analyze the structure of bioglass (Figure 2c). The FT-IR spectrum showed characteristic bending vibration of phosphate group peaks at 561 and 603 cm^−1^ and Si-O-Si stretching band at 1080 cm^−1^. After synthesizing BGN, its bioactivity postimplantation was tested. BGN was immersed for 14 days in simulated body fluid (SBF) solution and compared with prepared BGN by X-ray powder diffraction (XRD). As shown in Figure 2d, same hydroxyapatite peaks as those noted by Constantz et al. and Ishikawa et al. were presented in BGN soaked in SBF solution, confirming the bioactivity of synthesized BGN [43,44].

### 3.2. Characterization of Bioglass-Embedded Methacrylated Gelatin Cryogel

**Figure 3:**
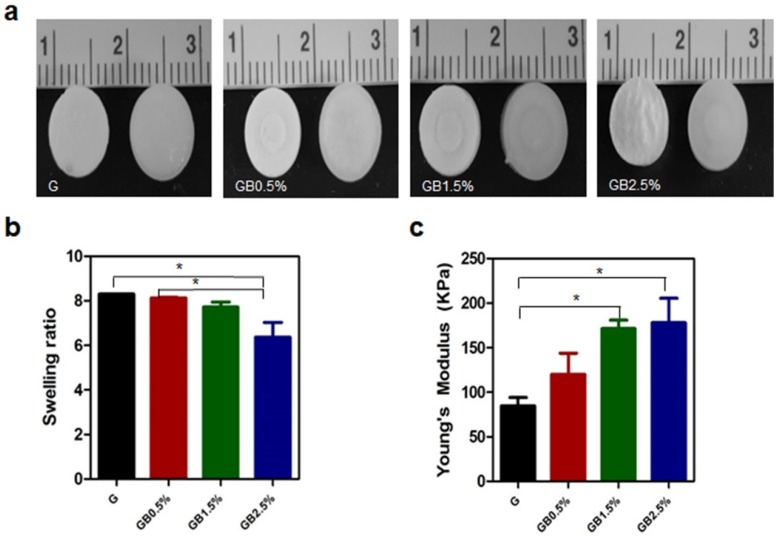
Scaffold characterization (**a**) Gross images of dried and swollen cryogels; (**b**) Swelling ratio; (**c**) Young’s modulus of cryogels (n = 3, * *p* < 0.05). Error bars indicate SD. G represents GelMA cryogel, and GB represents bioglass-incorporated GelMA cryogel with the concentration of bioglass noted.

GelMA cryogels were synthesized via chemically cross-linking GelMA solution using ammonium persulfate and *N*,*N*,*N*′,*N*′-tetramethylethylenediamine with various concentration of BGN (Figure 3a). Then, the swelling ratio of BGN-embedded GelMA cryogels was measured. As shown in Figure 3b, the descending trend of swelling ratio was observed from 8.30 to 6.37 as the concentration of bioglass in cryogels was increased. Furthermore, Young’s modulus of all groups was measured through analyzing stress vs. strain graph from the mechanical testing. As expected, Young’s modulus of cryogel was increased as the concentration of BGN also augmented from 84.67 ± 16.17 to 178 ± 47.70 kPa (Figure 3c).

The degradation rates of cryogel were tested for their dependency on the bioglass concentration in the presence of collagenase II. Collagenase II was widely used to test degradation times of GelMA-based cryogel [41,45]. The degradation time was not significantly different between the control group and the experimental group due to the relatively low concentration of BGN compared to that of GelMA (Figure S2). The remaining mass percentage after degrading GelMA cryogel for 7 days was 49.80 ± 2.91%, while remaining mass percentages of cryogels for 0.5%, 1.5%, and 2.5% BGN were 66.33 ± 8.73%, 60.48 ± 2.69%, and 64.02 ± 6.11%, respectively.

**Figure 4:**
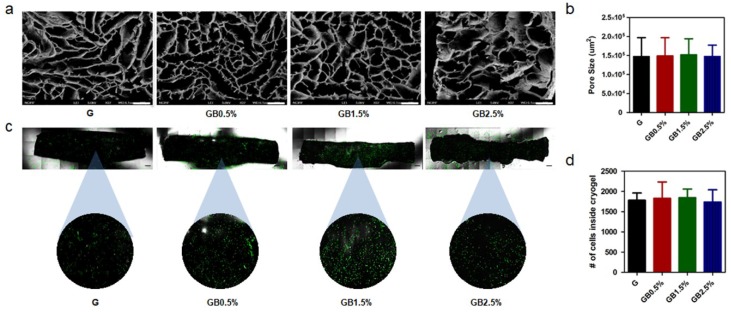
Internal structure of cryogels (**a**) Cross-sectional view of cryogels, as studied by FE-SEM; scale bar = 400 μm; (**b**) Average pore area of lyophilized cryogel with different bioglass concentration; error bars indicate SD (n = 3); (**c**) cell permeability in G, GB0.5%, GB1.5%, and GB2.5% cryogels; scale bar = 500 μm; (**d**) Number of cells inside the cryogel; error bars indicate SD (n = 3).

Because the increase in Young’s modulus seemed to be linked to the increase in bioglass concentration incorporated to cryogel, internal structures of cryogels were observed to ensure that the differences in porosity between experimental and control groups were negligible. The internal structures of BGN-embedded GelMA cryogels were observed using SEM (Figure 4a). Then, the SEM images of lyophilized BGN-embedded GelMA cryogels were analyzed further with ImageJ for average pore area. As shown in Figure 4b, there was no significant difference among the groups with different concentration of BGN. Since BGN was physically mixed with GelMA solution when cryogel fabrication was performed, both the experimental groups and the control group possessed similar internal structures. Furthermore, cell permeabilities of scaffolds were examined via seeding green fluorescent protein (GFP)-tagged HELA cells on GelMA and GelMA-bioglass cryogels and using confocal microscopy after 24 h (Figure 4c). Then, in order to quantify the cell permeabilities of scaffolds, the number of cells was counted and, expectedly, all cryogel groups had uniform cell permeability (Figure 4d).

### 3.3. Hydroxyapatite Formation in SBF Solution

**Figure 5:**
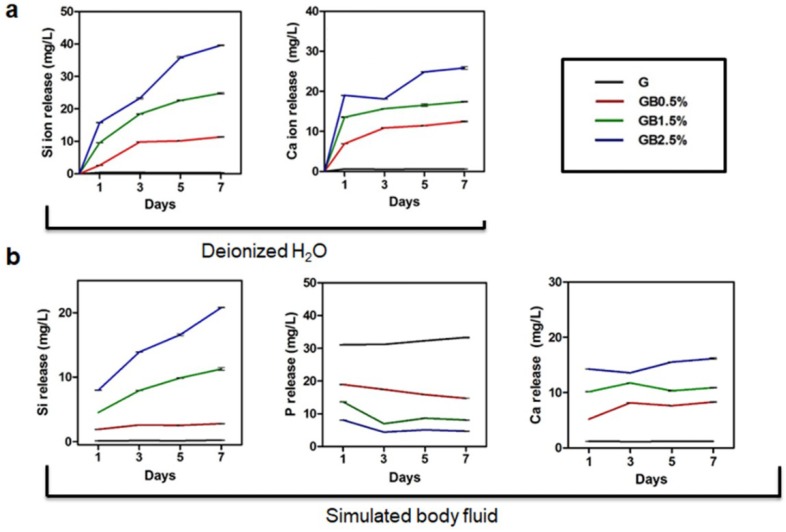
Ion release analysis. Ion dissolution rates of cryogels tested for period of seven days in (**a**) deionized water and (**b**) simulated body fluid; error bars indicate SD.

Various studies have confirmed the bioactivity of intact bioglass nanoparticle; yet, bioactivities of cryogels in which BGN are embedded have not been emphasized. First, in order to measure Si^4+^ and Ca^2+^ ion release from bioglass-embedded cryogels, BGN-embedded cryogels were incubated in deionized water for 7 days and measured using inductively coupled plasma atomic emission spectrometer. As shown in Figure 5a, as the concentration of nanobioglass in cryogel increased, released amount of Si^4+^ and Ca^2+^ ion increased as well. To measure the bioactivity, BGN-embedded cryogels were incubated in SBF solution for 7 days, and phosphorus and silicon ion release kinetics were measured. Based on the incubation time, the amount of released silicon ions increased as the amount of released phosphorus ions decreased (Figure 5b).

**Figure 6:**
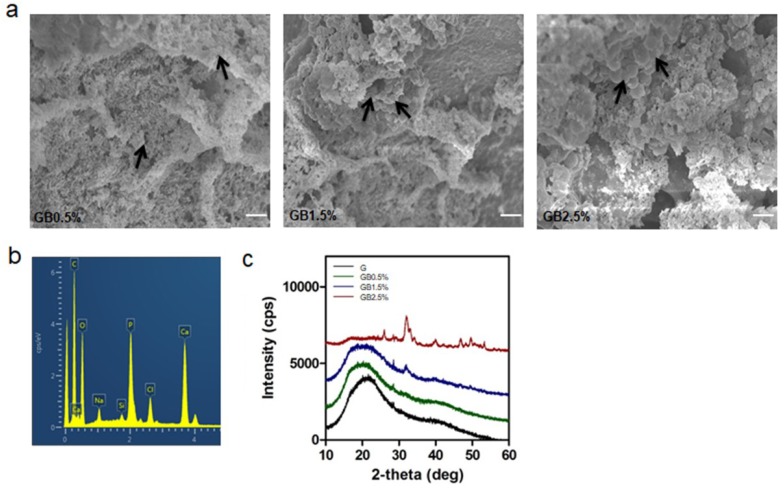
Bioactivity of bioglass (**a**) SEM images of GB0.5%, GB1.5%, and GB2.5% cryogels after immersing in SBF solution for seven days. Black arrow represents formed hydroxyapatite; scale bar = 1 μm; (**b**) EDS mapping of GB2.5%; (**c**) XRD of G, GB0.5%, GB1.5%, and GB2.5% cryogels.

After seven days of incubation, internal structures of each group were observed through SEM for possible hydroxyapatite formation. As shown in Figure 5a, were able to find formed hydroxyapatite particles from SEM image (Figure 6a). Furthermore, calcium to phosphate ratio on the surfaces of cryogels was calculated to be 1.63, which was very close to the textbook value of calcium to phosphorus ratio of hydroxyapatite (Figure 6b). Then, the relationship between the formed hydroxyapatite and concentration of BGN was analyzed with XRD. Figure 6c shows that the intensity of hydroxyapatite concentration increased as the concentration of BGN increased. Among the groups, 2.5% BGN-embedded cryogel resulted in the highest intensity.

### 3.4. Cell Viability Analysis

**Figure 7:**
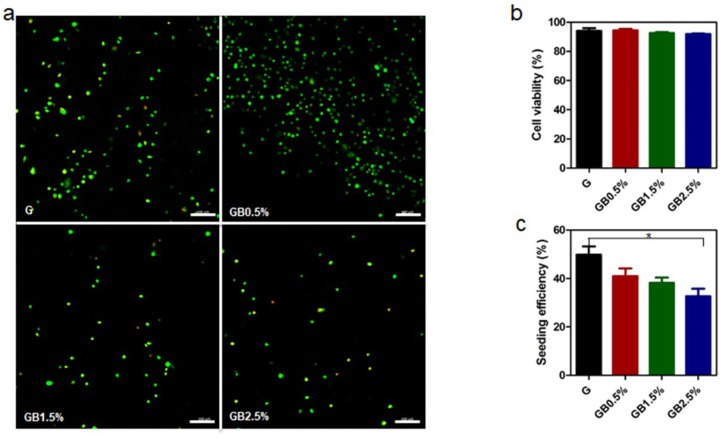
Cell viability test: (**a**) Fluorescence images of live/dead staining of human tonsil-derived stem cells (hTMSCs) cultured for 24 h on GelMA and bioglass nanoparticles-incorporated cryogels; scale bar = 100 μm; (**b**) cell viability as quantified by live/dead assay using ImageJ; error bars indicate SD (n = 3); (**c**) seeding efficiency of hTMSCs that were seeded on GelMA and bioglass nanoparticles-incorporated cryogels; error bars indicate SD (n = 3).

Cell viability tests were performed to investigate the cytotoxicity of groups for further cellular responses. Human tonsil-derived mesenchymal stem cells were seeded on BGN-embedded cryogels and after 24 h of incubation, live/dead viability test was proceeded. Fluorescence images of each group after incubating (hTMSCs) on cryogels for 24 h were observed (Figure 7a). Then, cell viabilities of each group were calculated based on fluorescence images. The percentage of cell viability was slightly decreased as the concentration of bioglass increased; however, all groups showed over 90% of cell viability (Figure 7b). For the seeding efficiency of hTMSCs on GelMA and bioglass nanoparticles incorporated cryogels, the percentage of seeding efficiency decreased as the concentration of bioglass increased (Figure 7c). This indicates that increase of bioglass concentration lead less cells to be seeded on the surface of GelMA. However, all groups showed over 30% of cell seeding efficiency.

### 3.5. Enhanced Osteogenic Responses on hTMSCs on GelMA-Bioglass cryogel

**Figure 8:**
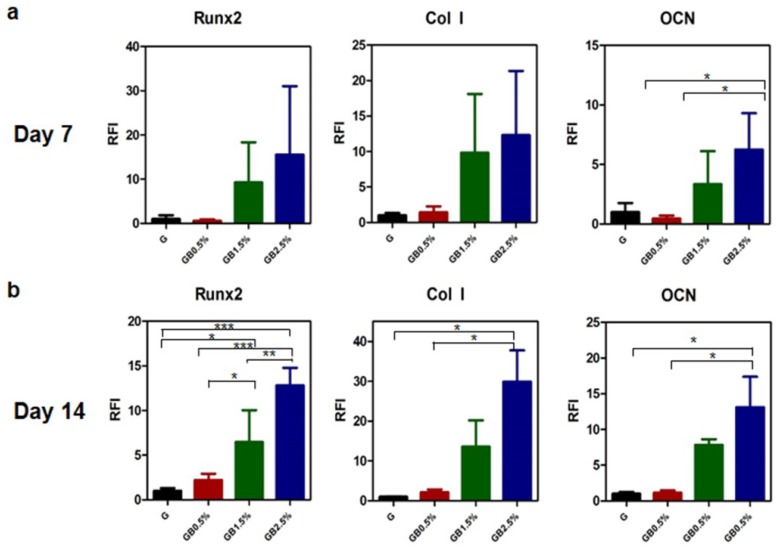
Relative osteogenic gene expression. Relative osteogenic expression of hTMSCs seeded on cryogels (**a**) at day 7, (**b**) at day 14 (n = 3, * *p* < 0.05, ** *p* < 0.001, *** *p* < 0.0001); error bars indicate SD.

After confirming hydroxyapatite formation and cytotoxicity, osteogenic potential of BGN-embedded cryogels was verified by seeding hTMSCs on top of cryogels and culturing the cells with osteogenic medium for 7 and 14 days to measure relative osteogenic gene expression. On both day 7 and day 14, quantitative real-time PCR analysis of osteogenic markers confirmed that hTMSCs seeded on cryogels with higher concentration of bioglass (1.5% and 2.5% of BGN) augmented bone-related gene expressions compared to GelMA cryogel at day 14—7.84 (1.5% BGN) and 13.13 (2.5% BGN) fold increase in *osteocalcin*, 13.56 (1.5% BGN) and 29.92 (2.5% BGN) fold increase in *collagen I*, and 6.47 (1.5% BGN) and 12.83 (2.5% BGN) fold increase in *Runx2* (Figure 8). However, the difference in gene expressions of GelMA cryogel and GelMA-0.5% bioglass cryogel was not significant on day 7 and day 14.

Further investigating cellular responses of hTMSCs, the cells were cultured on petri dishes in osteogenic medium with similar concentration of bioglass for 21 days. Then, the amount of calcium deposition of each sample was measured using alizarin red staining (Figure S3). The result was similar to that of quantitative real-time PCR—higher calcium deposition was observed as bioglass dosage increased.

### 3.6. In Vivo Bone Regeneration after Implanting Bioglass-Embedded GelMA Cryogel

**Figure 9:**
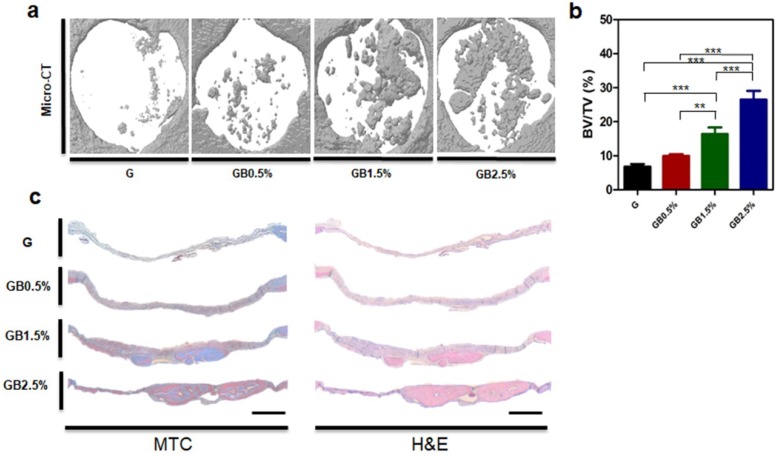
In vivo experiment. (**a**) microCT images of cranial defect (defect diameter = 4 mm) and (**b**) normalized bone volume/ total volume (BV/TV) of defect after eight weeks of transplantation; error bars indicate SD (n = 3). (**c**) Histological analysis of Masson Trichrome and H&E staining; scale bar = 500 μm.

To examine the optimal concentration of BGN embedded in GelMA cryogels for effective bone regeneration, in vivo studies were conducted. GelMA-bioglass cryogels were implanted to calvarial defect areas of balb/c mice. Then, bone regeneration of the defected areas was evaluated using microCT eight weeks after transplantation (Figure 9a). Corresponding to in vitro cell studies, bone volume/ total volume (BV/TV) of cryogels containing the highest bioglass concentration was 3.89-fold higher compared to the control group (Figure 9b). In addition, cell penetration on the scaffold was evaluated via histology. Histological analysis of H&E staining and Masson’s trichrome staining (MTC) showed that as the concentration of bioglass increased, more regenerated bone tissues along with collagen were found in the defect area where the nanobioglass-embedded cryogels were implanted (Figure 9c). Histological analysis and microCT confirmed that GelMA-2.5% bioglass cryogel enhanced bone regeneration the most compared to other experimental groups.

## 4. Discussion

Synthetic calcium phosphate-based materials have been widely studied for regenerating defected bone area. Among them, bioglass is valued for its ability to enhance osteogenic differentiation by forming hydroxyapatite and releasing osteoinductive ions; various studies have demonstrated the invaluable effects of osteoinductive ions on bone regeneration process [46,47,48,49,50]. In addition to supplying calcium phosphate, providing extracellular matrix-like environment enhances osteoregeneration during the bone healing process [51,52]. Because it is capable of mimicking natural ECM and has a porous structure, cryogel is widely used in tissue engineering [53,54]. From this idea, we embedded bioglass into GelMA cryogels in an attempt to provide an osteogenic-friendly environment (Figure 1). Furthermore, the macroporous structure of GelMA would provide cancellous bone-like structure where host stem cells can migrate. A closer look at the microstructure of GelMA-based cryogel showed that the overall pore size was unaffected by the addition of bioglass (Figure 4a). However, physical adhesion between GelMA and bioglass may have contributed to the bioglass concentration-dependent reduction in swelling ratio and increase in mechanical properties of cryogel. Pluharova et al. have previously demonstrated that ion-dipole interaction persists between amide group and calcium ions [55]. Thus, this physical adhesion in combination with ion-dipole interactions from calcium ions from bioglass and amide groups in GelMA may have contributed to the overall physical characteristics of cryogels.

Mineralization of inorganic minerals plays an important role in maintaining bioactivities in body fluids [56,57]. The mechanism of surface chemistry and bioactivity of bioglass powder in SBF solution has been widely studied [58,59]. From the results obtained in this study via testing bioactivities of bioglass-embedded cryogels, formation of hydroxyapatite was observed via XRD. However, the ion concentration threshold seemed to be present according to XRD. The amount of mineral deposited was gradually increased with the concentration of bioglass until 1.5% and sharp increase with 2.5% bioglass concentration (Figure 6c). This result suggested that the minimum amount of bioglass was required to trigger hydroxyapatite formation in early periods since the rate of hydroxyapatite formation is depended on presence of Ca^2+^ and P^3+^ ions. This result is similar to the findings of Hench et al. as the authors discovered that the rate of hydroxyapatite formation is depended on concentration of bioglass [60]. 

Ion release rates of Si^4+^, Ca^2+^, and P^3+^ ions from bioglass immersed in SBF solution revealed that Si^4+^ ions were released to form silanol, enhancing overall hydroxyapatite formation by forming apatite nuclei (Figure 5) [61]. After forming apatite nuclei, the saturation of Ca^2+^ ions was reached in the solution. Then, the hydroxyapatite layer was formed via recapturing and adsorbed P^3+^ ions, which were released to the solution on the apatite nuclei. Thus, the concentrations of P^3+^ ions decreased as time passed.

Furthermore, the mineralized surfaces of cryogels were measured via EDS mapping, and only the GelMA-2.5% bioglass group showed apatite with 1.63 Ca^2+^/P^3+^ ratio; this is similar to the theoretical Ca^2+^/P^3+^ value of 1.67 (Figure 6b). This result suggests a possible direct cryogel-bone bonding, which may increase the in vivo bone generation [62].

Osteogenic effects of bioglass-embedded GelMA cryogels on hTMSCs were measured using RT-PCR and alizarin red staining. In our studies, osteogenic genes—*OCN*, *Runx2*, and *Col I*—were further up-regulated according to the concentration of bioglass on day 7 and day 14 (Figure 8), and similar trend was observed when calcium deposition was measured after 21 days via alizarin red staining (Figure S3). Osteogenic differentiation of hTMSCs was enhanced due to ion dissolutions from bioglass in osteogenic medium since similar trend of Ca^2+^ and Si^4+^ ion release rates were observed according to bioglass dosage embedded in cryogel (Figure 5 and Figure 8). Calcium ion released from bioglass enhanced mineralization, inducing guiding stem cells to osteoblasts [63]. Furthermore, S. Maeno et al. also supported that Ca^2+^ ion induces osteogenic differentiation in both monolayer and 3D culture [64]. Our study confirmed that osteogenic differentiation of hTMSCs is dependent on the concentration of bioglass incorporated in cryogel. Si^4+^ ion—a bioglass component—is well known to promote bone formation and calcification in the early stages, though high concentrations of Si^4+^ ion increase cytotoxicity. In our studies, large amounts of silicon ion had not been incorporated into bioglass; however, as expected, cryogel with the highest concentration of bioglass showed the most cytotoxicity among the groups (Figure 7b). All groups showed over 90% of cell viability, which suggests that although increased concentration of Si^4+^ leads to higher cytotoxicity, it would be safe to use for the concentration that was used in this study.

Based on in vitro results, cryogels were implanted for eight weeks and measured to examine if bone regeneration in a calvarial defect improved. As demonstrated by in vitro results, bioglass helped bone regeneration of defected area. According to microCT data, cryogels with the highest concentration of bioglass nanoparticles had the most bone healing compared to other cryogel groups (Figure 9). Furthermore, higher concentration of collagen was observed via Masson’s trichrome staining and H&E staining in the highest bioglass concentration group. In conclusion, bioglass has an osteoinductive effect in bone healing process.

## 5. Conclusions

From our study, we demonstrated that bioglass-incorporated GelMA cryogels effectively promoted bone healing. Tested concentrations of bioglass-embedded cryogel showed enhanced mechanical strength without affecting the porosity and cytotoxicity. Furthermore, it not only induced osteogenic differentiation of hTMSCs but was also bioactive, forming hydroxyapatite on its surface. Thus, by manipulating the dose-dependent nature of bioglass, bioglass-incorporated cryogel could be a potential candidate in clinical application for bone tissue engineering.

## Supplementary Materials

The following are available online at http://www.mdpi.com/2073-4360/10/8/914/s1, Figure S1: ^1^H NMR of gelatin and methacrylated gelatin, Figure S2: Degradation rates of cryogel using collagenase II solution for 7 days, Figure S3: Alizarin red staining after 21 days.
