# Supplementary material for: Bioglass-Incorporated Methacrylated Gelatin Cryogel for Regeneration of Bone Defects
Source: Polymers (Basel). 2018 Aug 14;10(8):914. doi: 10.3390/polym10080914 (PMC6403913; doi:10.3390/polym10080914)
Supplement: Supplementary file 1 [file polymers-10-00914-s001.docx]

Supporting Information for Publication

Bioglass Incorporated Methacrylated Gelatin Cryogel for Regeneration of Bone Defects

*Song Kwon^1,‡^, Seunghun S. Lee^2,‡^, A. Sivashanmugam^3^, Janet Kwon^2^, Seung Hyun L. Kim^2^*, *Mi Yeon Noh^2^,* *R. Jayakumar^3^ and Nathaniel S. Hwang^1,2,4,*^*

^1^ School of Chemical and Biological Engineering, the Institute of Chemical Processes, Seoul National University, Seoul, 08826, Republic of Korea

^2^ Interdisciplinary Program in Bioengineering, Seoul National University, Seoul, 08826, Republic of Korea.

^3^ Center for Nanosciences and Molecular Medicine, Amrita Institute of Medical Sciences and Research Center, Amrita Vishwa Vidyapeetham, Kochi, 682041, India

^4^ BioMAX/N-Bio Institute, Seoul National University, Seoul, 08826, Republic of Korea

‡ Contributed equally to this work.

^*^To whom the correspondence should be addressed:

Prof. Nathaniel Hwang, Ph.D.

School of Chemical and Biological Engineering

Seoul National University

+82-2-880-1635

nshwang@snu.ac.kr

**Supporting Information Captions**

**Figure S1.** ^1^H NMR of gelatin and methacrylated gelatin

**Figure S2.** Degradation rates of cryogel using collagenase II solution for 7 days

**Figure S3.** Alizarin red staining after 21 days

**
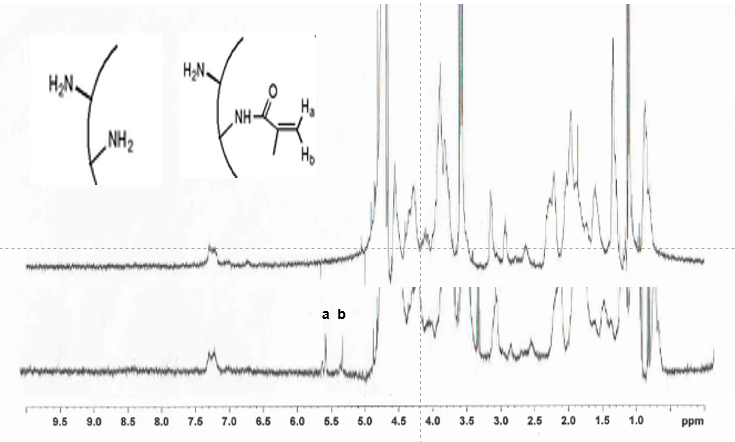
**

**Figure S1.** ^1^H NMR of gelatin and methacrylated gelatin

**
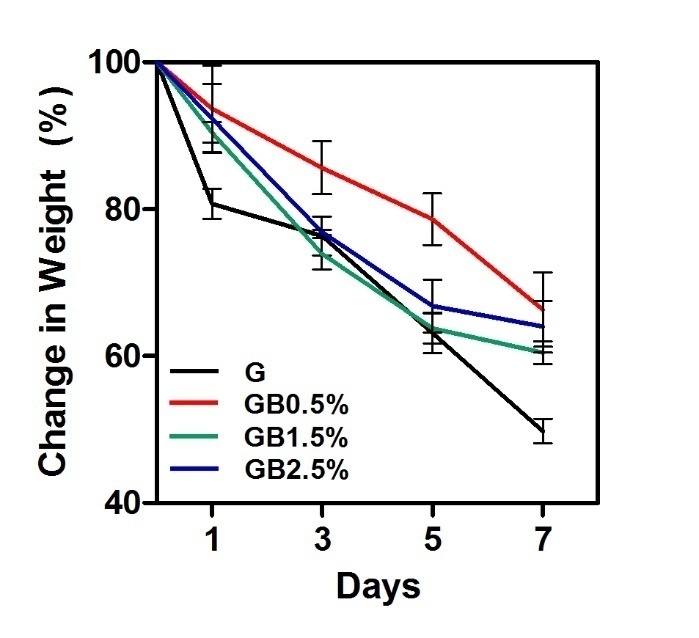
**

**Figure S2.** Degradation rates of cryogel using collagenase II solution for 7 days. Error bars indicate SD.


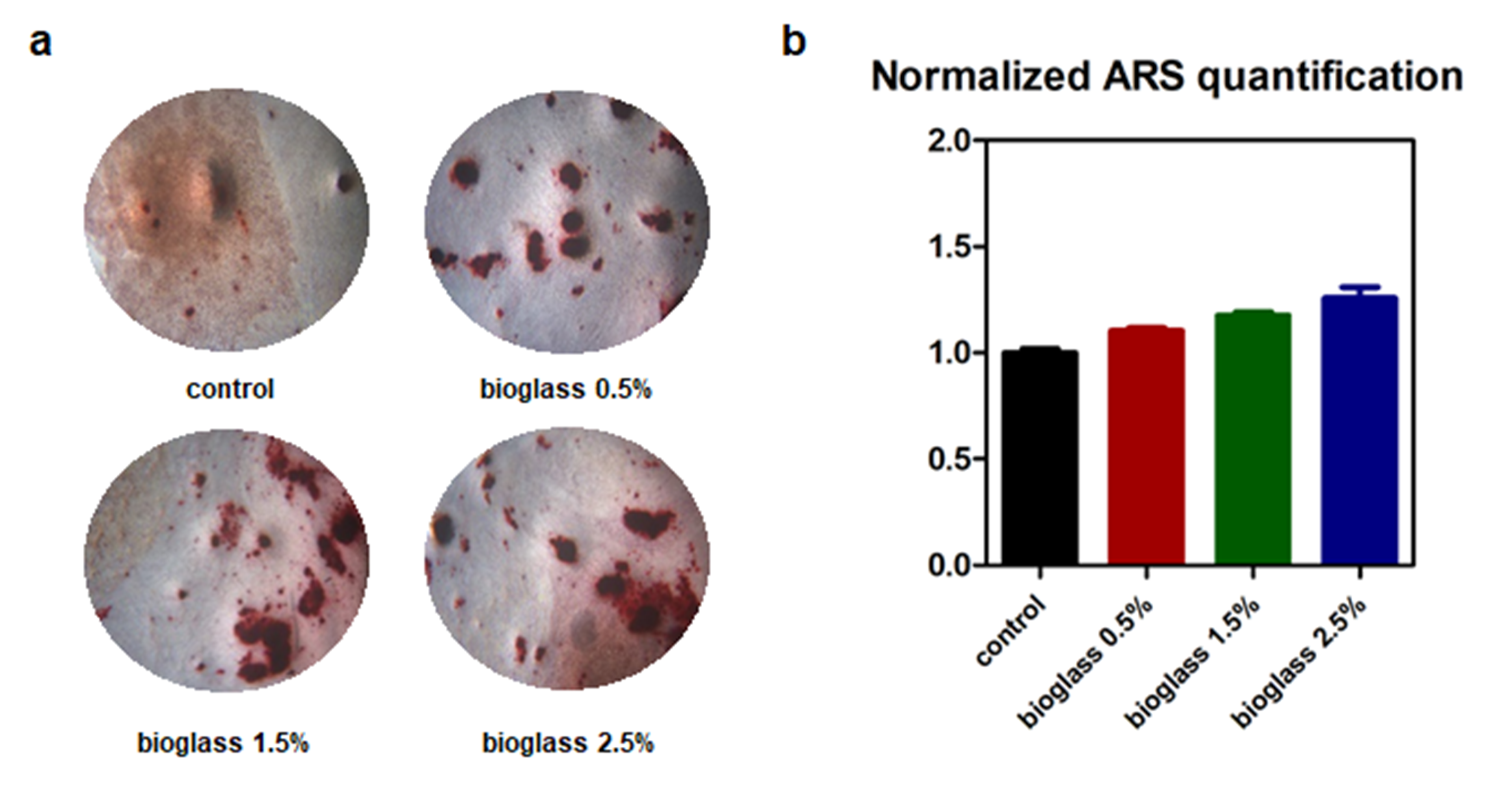


**Figure S3.** Alizarin red staining after 21 days. Control group was cultured with just osteogenic medium (OM) while other groups were cultured with OM and bioglass concentration (0.5, 1.5 and 2.5%) Error bars indicate SD. (n = 3)
